# A Tale of Thirty Voyeurs – A Case Series Detailing Psychological Profiles and Risk Factors for Voyeuristic Sexual Offending in Singapore

**DOI:** 10.1192/bjo.2026.11131

**Published:** 2026-06-30

**Authors:** Keng Chuan Soh, Razwana Begum Bt Abdul Rahim, Rosleenda Binte Mohamed Ali, Derrick Chen Kuan Yeo, Christopher Cheng Soon Cheok

**Affiliations:** 1 Institute of Mental Health, Singapore, Singapore; 2 Singapore University of Social Sciences, Singapore, Singapore

## Abstract

**Aims:**

Voyeurism represents a significant public safety concern in Singapore, ranking third amongst crimes of concern with a 9% increase in cases from 2023 to 2024. Despite its prevalence, limited research exists on the psychological profiles and risk factors of individuals engaging in voyeuristic behaviour in local contexts.

This study seeks to develop a foundational understanding of potential patterns and risk factors for voyeuristic acts in Singapore’s local context. It postulates that there are predictable, recurring patterns for how individuals engage in voyeuristic acts, with risk factors such as social isolation, dysfunctional interpersonal relationships, the lack of a meaningful romantic relationship, antisocial personality traits, and concurrent mental illness.

**Methods:**

A retrospective medical records review was conducted of cases known to the Department of Forensic Psychiatry at the Institute of Mental Health, Singapore.

The study was approved by the National Healthcare Group Domain Specific Review Board, which granted waiver of informed consent (reference number 2024-4055). The study uses de-identified data in a secure manner that would not pose risk to study subjects.

Sample Selection: Records were reviewed for individuals who had engaged in voyeuristic acts, either allegedly or confirmed through legal conviction, between January 2023 and December 2024. No age restrictions were applied. These subjects presented to the department through one or more of the following routes: remand assessments, suitability for Mandatory Treatment Order, or follow-up after their release from prison.

Data Collection: Data was extracted from medical records covering their demographics, offending patterns, relationship history, social factors, mental health diagnoses, substance use, criminal history, and treatment history. Data was de-identified by an independent party before analysis to ensure confidentiality.

**Results:**

All 30 subjects were male (median age 28.5 years), with 73.3% having tertiary education. Social isolation was prevalent (68.8% of those with available data), alongside significant family dysfunction. The median number of voyeuristic acts was 3.5 per individual. Offending patterns were quite evenly divided between upskirting and toilet/shower voyeurism. Mental health diagnoses were present in 73.3% of participants, with Voyeuristic Disorder being most common (23.3%). Antisocial traits were rare, while substance use was virtually absent.

**Conclusion:**

Voyeuristic behaviour in this clinical sample was characterised by interpersonal dysfunction rather than traditional antisocial risk factors. The high educational attainment and low antisocial traits suggest this represents a distinct subgroup of help-seeking individuals. Treatment interventions should prioritise addressing underlying interpersonal difficulties and attachment dysfunction rather than focusing solely on traditional risk factors.

